# The BCG Vaccine for COVID-19: First Verdict and Future Directions

**DOI:** 10.3389/fimmu.2021.632478

**Published:** 2021-03-08

**Authors:** Maria Gonzalez-Perez, Rodrigo Sanchez-Tarjuelo, Boris Shor, Estanislao Nistal-Villan, Jordi Ochando

**Affiliations:** ^1^Transplant Immunology Unit, Department of Immunology, National Center of Microbiology, Instituto De Salud Carlos III, Madrid, Spain; ^2^Department of Oncological Sciences, Icahn School of Medicine at Mount Sinai, New York, NY, United States; ^3^Manhattan BioSolutions, New York, NY, United States; ^4^Microbiology Section, Departamento de Ciencias Farmacéuticas y de la Salud, Facultad de Farmacia, Universidad San Pablo-Centro de Estudios Universitarios (CEU), Madrid, Spain; ^5^Facultad de Medicina, Instituto de Medicina Molecular Aplicada (IMMA), Universidad San Pablo-CEU, Madrid, Spain

**Keywords:** Bacille Calmette-Guerin, SARS-CoV-2, vaccination, trained immunity, cross-protection

## Abstract

Despite of the rapid development of the vaccines against the severe acute respiratory syndrome coronavirus 2 (SARS-CoV-2), it will take several months to have enough doses and the proper infrastructure to vaccinate a good proportion of the world population. In this interim, the accessibility to the Bacille Calmette-Guerin (BCG) may mitigate the pandemic impact in some countries and the BCG vaccine offers significant advantages and flexibility in the way clinical vaccines are administered. BCG vaccination is a highly cost-effective intervention against tuberculosis (TB) and many low-and lower-middle-income countries would likely have the infrastructure, and health care personnel sufficiently familiar with the conventional TB vaccine to mount full-scale efforts to administer novel BCG-based vaccine for COVID-19. This suggests the potential for BCG to overcome future barriers to vaccine roll-out in the countries where health systems are fragile and where the effects of this new coronavirus could be catastrophic. Many studies have reported cross-protective effects of the BCG vaccine toward non-tuberculosis related diseases. Mechanistically, this cross-protective effect of the BCG vaccine can be explained, in part, by trained immunity, a recently discovered program of innate immune memory, which is characterized by non-permanent epigenetic reprogramming of macrophages that leads to increased inflammatory cytokine production and consequently potent immune responses. In this review, we summarize recent work highlighting the potential use of BCG for the treatment respiratory infectious diseases and ongoing SARS-CoV-2 clinical trials. In situations where no other specific prophylactic tools are available, the BCG vaccine could be used as a potential adjuvant, to decrease sickness of SARS-CoV-2 infection and/or to mitigate the effects of concurrent respiratory infections.

## Introduction

Tuberculosis (TB) is a respiratory disease caused by *Mycobacterium tuberculosis* complex (1), a group of bacteria which primarily attacks the lungs along with other body parts such as the kidneys, spine, and brain. This airborne infectious disease can spread from person to person and it was the leading cause of death in the 1900's in Europe. Following 20 years of research the TB vaccine was developed by Albert Calmette and Camille Guérin, from whose initials takes its name (BCG, Bacillus Calmette-Guerin). Obtaining the vaccine was not an easy procedure. Calmette and Guérin cultivated *Mycobacterium bovis*, one of the possible tubercle bacilli on a glycerin and potato culture medium and subsequently added ox bile to obtain a more homogeneous suspension, which ended up lowering the virulence of the bacterium. The result was an attenuated strain of the bacteria that could be used as a vaccine. They decided to carry out several animal trials (guinea pigs, rabbits, or horses) until in 1921 they first administered BCG to humans at the Charité Hospital, Paris. From that on, 114.000 infants were orally vaccinated without serious complications until 1928 and the BCG vaccination was proved to be safe (2).

It was soon after its administration that it was observed the beneficial effects of BCG vaccination, with multiple studies reporting non-specific cross-protection of the vaccine against other infectious diseases. Carl Näslund introduced the BCG as a prevention of TB in northern Sweden and informed in 1932 that the vaccine was reducing child mortality unrelated to the TB disease, suggesting that “one could evidently be tempted to find an explanation for this lower mortality among vaccinated children in the idea that BCG provokes a non-specific immunity” (3). However, these protective effects were not studied in detail until nearly 70 years after, when Peter Aaby et al. first reported a 45% mortality reduction due to non-specific beneficial effects of BCG vaccination in West Africa. These pioneer studies described that BCG vaccination was associated with lower incidence of neonatal sepsis and respiratory tract infections that reduced child mortality (4, 5). Further, Peter Aaby et al. reported 60% less acute lower respiratory tract infection (ALRI) in girls below 5 years of age, confirming a non-targeted beneficial effect of BCG vaccination on childhood survival (6). Other examples of BCG vaccination mediated non-specific effects include 70% reduction in pneumonia in Japan (7); 80% decrease in acute upper respiratory tract infection (AURTI), including rhino-pharyngo-laryngo-tracheitis, in the elderly (60–75 years old) in Indonesia (8); and 73% lower rate in upper respiratory tract infections in a phase 2 trial conducted in South Africa (CT02075203) (9). More recently, it has been stablished that BCG vaccination protects against a variety of viral infections such as influenza virus, yellow fever virus, herpes simplex viruses, respiratory syncytial virus, and human papilloma virus (10).

Since January 2020, a pandemic situation has emerged worldwide as a result of a novel severe acute respiratory syndrome coronavirus 2 (SARS-CoV-2) outbreak. The virus infects the respiratory tract and replicates in cells expressing the ACE2 receptor that are present in the lung parenchyma pneumocytes, the goblet cells along the nasal mucosa and in absorptive enterocytes in the small intestine (11). Scientists around the world are continuously working to develop specific vaccines to lower the incidence of COVID-19 in the population. As there is an urgent need to mitigate the effects of COVID-19, governmental and non-governmental agencies, including the World Health Organization (WHO), urged to test approved therapies that may be used to reduce the consequences of SARS-CoV-2 infection, such as drugs developed for AIDS, Ebola, or Malaria, excluding BCG vaccination, as there was no evidence that the BCG vaccine protected people against infection (12). As of February 2021, several vaccines have been approved for therapeutic use. These include two messenger RNA-based vaccines (Pfizer-Biotec and Moderna) and one adenovirus-based attenuated vaccine (Oxford-AstraZeneca). The three RNA vaccines have been approved by the American Food and Drug Administration (FDA) and the European Medicines Agency (EMA) (13, 14) and are being currently administered to millions of people. In addition, the Russian vaccine Sputnik has been licensed in Russia, Belarus, Hungary, Servia, United Arab Emirates and Argentina, and more than 50 countries in Latin America Europe, Africa, and Asia have purchased the vaccine, while the Chinese vaccines CoronaVac (15) and Sinopharm have been approved for emergency used in China and in the United Arab Emirates, respectively.

In principle, the arrival of SARS-CoV-2 specific vaccines and initial clinical trial results concluding that BCG does not reduce COVID-19 mortality overrides the need to further evaluate the effects of BCG vaccination on SARS-CoV-2 infection. However, the BCG vaccine may represent a therapeutic approach to (i) increase the efficacy of current and future SARS-CoV-2 specific vaccines that aim at developing immunological memory, while (ii) mitigate the effects of potential concurrent infections. BCG vaccination offers certain level of protection against different respiratory viral, bacterial and fungal infections that may coexist with SARS-CoV-2 (16) and therefore, coadministration of BCG and SARS-CoV-2 vaccines may have synergistic protective effects, including increased efficacy and/or duration of the memory response. In addition, since BCG vaccination might be associated with a decrease in the incidence of sickness during the COVID-19 pandemic (17), it may help to reduce hospitalizations dure to SARS-CoV-2 and other respiratory infections. Here, we review some recent data that addresses the potential use of BCG to diminish the magnitude of COVID-19 and other acute respiratory diseases.

## BCG, Trained Immunity and Crossprotection

**Figure 1:**
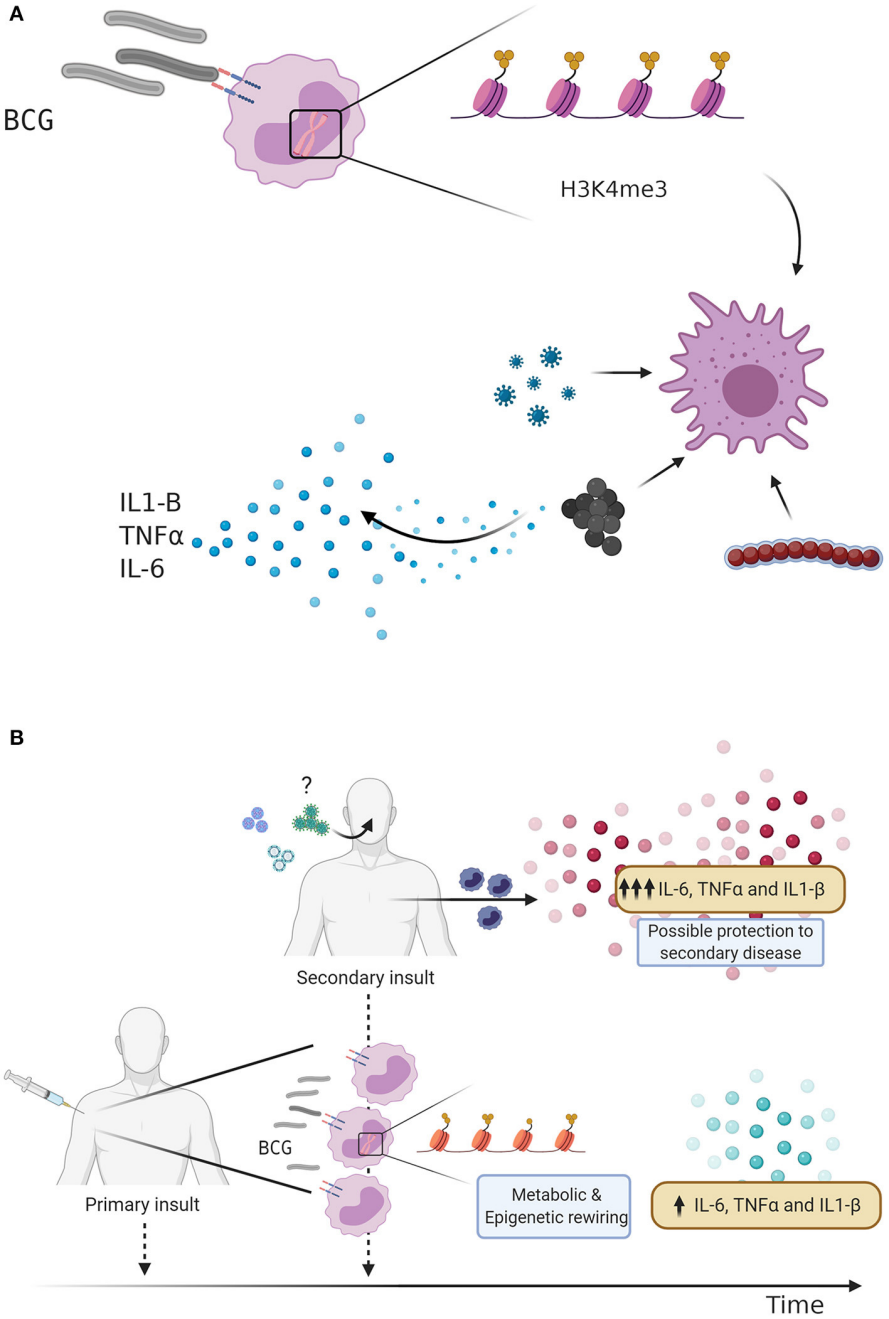
**(A)** BCG vaccination and Trained immunity. Bacillus Calmette-Guérin (BCG) vaccination induces trained immunity through metabolic changes and epigenetic rewiring. Trimethylation of the H3K4 histone predisposes the innate immune response to a secondary insult, leading to an increased production of pro-inflammatory cytokines. **(B)** Trained immunity as defense mechanism against respiratory infections. BCG vaccination is given as an initial stimulus, leading to metabolic changes and epigenetic rewiring of innate immune cells, increasing the transcription of pro-inflammatory genes and secretion cytokines. BCG vaccinated individuals display an enhanced innate immune response following a secondary challenge, which may lead to protection against subsequent viral infections such as Influenza A virus or Respiratory syncytial virus (RSV). Could BCG vaccination also protect against severe acute respiratory syndrome coronavirus-2 (SARS-CoV-2)?

The ancient innate immune system has evolved to employ multiple defense mechanisms to eliminate infection. In contrast to the adaptive immunity, which relies on the antigen-specificity, additional innate immune cell populations may exhibit heterologous memory responses triggered upon microbial exposure. Indeed, several studies demonstrated that macrophages and natural killer (NK) cells, which have experienced previous pathogen encounter, can be “trained” via epigenetic remodeling to respond to unrelated pathogens (18, 19). Epigenetic marks including as H3K4me1, H3K4me3, and H3K27ac, mediate epigenetic reprogramming of monocytes which results in the opening of chromatin sites at the promoters of pro-inflammatory cytokines such as IL-6, IL-1β, and TNFα (20) (Figure 1). It is plausible that a cross-talk between macrophages and NK cells facilitates the fine-tuning of innate immune program during the life-time exposure to microbial insults. In support of this notion, vaccination of healthy volunteers with BCG primes macrophages and NK cells, leading to increased cytokine production after *ex vivo* restimulation (3, 21).

A recent study from Netea et al. demonstrated that the BCG vaccine offers a certain degree of cross-protection against a viral infections through trained immunity related mechanisms. Specifically, the study demonstrated the effects of BCG vaccination on genome-wide histone modifications induced in trained monocytes, which are associated with reduced levels of yellow fever virus (YFV) viremia due to increased IL-1β production and release (22). This cross-protection effects of BCG against YFV infection confirms the non-specific effects of BCG vaccine described for various viral infections, such as influenza A (H1N1), herpes virus (HSV), respiratory syncytial virus (RSV), and the human papilloma virus (HPV) (10). Since BCG offers protection to TB unrelated viral infections, it was hypothesized that BCG vaccination could also offer protection against SARS-CoV-2 infection in some individuals that have initial defective antiviral responses (23). To explain this, there is another type of immunological mechanism by which BCG could be inducing cross-protection named heterologous immunity. The term heterologous immunity refers to the immunity that can develop to one pathogen after an individual has been exposed to a non-identical pathogen. In this respect, a recent study addressing the homology between SARS-CoV-2 envelope protein and different *Mycobacterium* strains, presents one sequence of 12 amino acids of SARS-CoV-2 envelope protein has high homology to LytR C-terminal domain-containing proteins of *Mycobacterium* sp. (24). BLAST analysis is accompanied by detection of Mycobacteria in formalin and paraffin embedded tissue using immune-based microscopic assays showing a coincidental signal using acid-fast bacillus (AFB) staining and a SARS-CoV-2 envelope-specific antibody. In this manuscript, it is proposed that homology may activate heterologous immunity and a Th1/Th17 response, as previously described between adenovirus vaccine vectors and hepatitis C virus (HCV). Therefore, a combination of trained immunity and heterologous immunity by some BCG epitopes could provide immunity through T-cell cross-reactivity that could be responsible for the beneficial clinical effects of BCG.

## BCG Strains

**Figure 2:**
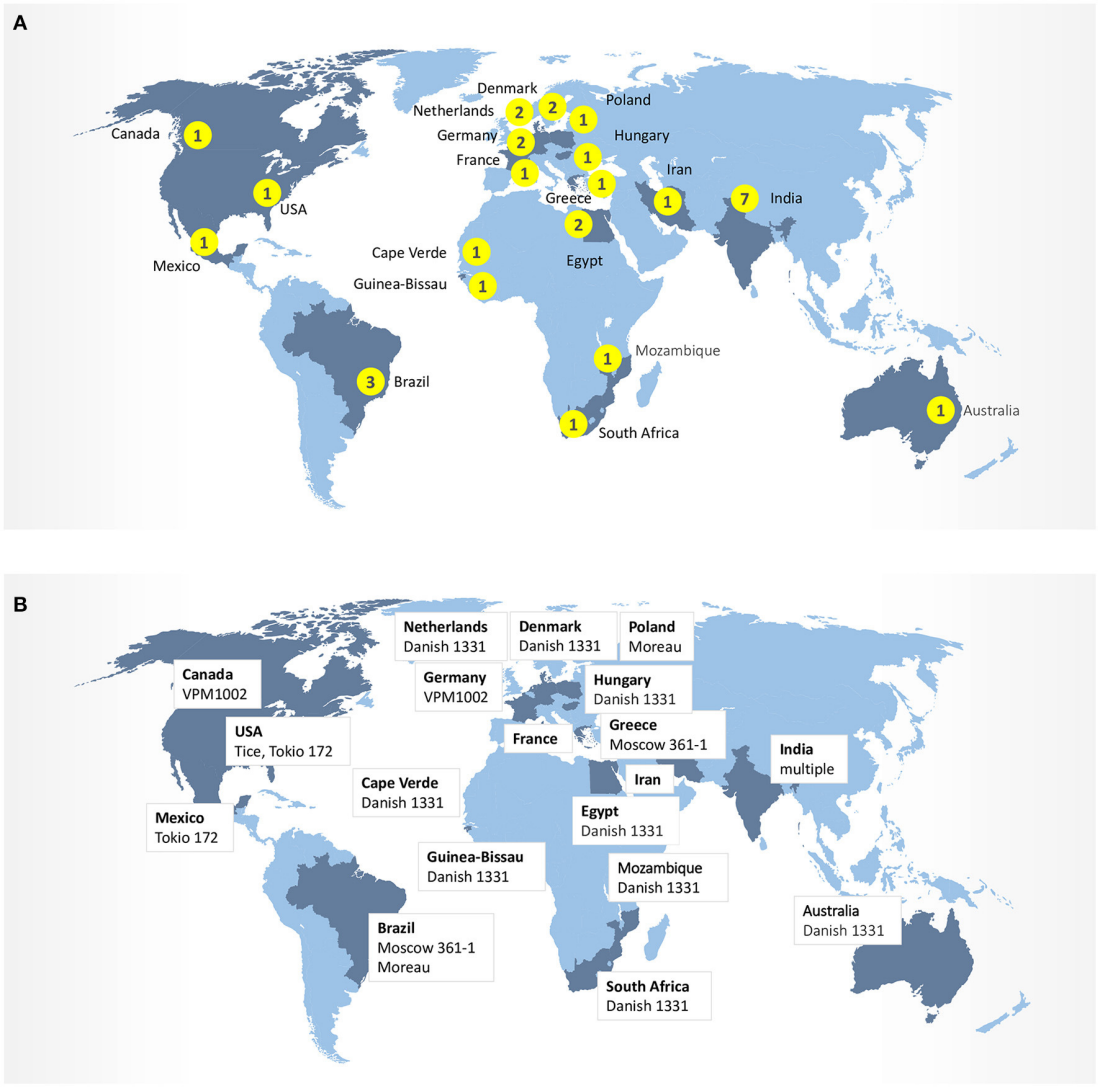
**(A)** Number of clinical trials BCG-COVID by country. **(B)** BCG strains in ongoing clinical trials.

In addition to trial design, the heterogeneity of BCG strains may also influence safety and effectiveness of vaccines against respiratory infections. Today, over 14 sub-strains of BCG have evolved and have been used as BCG vaccine in different laboratories around the world (Figure 2). It was suggested that the strain variation may contribute to the highly variable protective efficacy of BCG against TB observed in clinical trials. The comparative genomic analysis studies and more recently conducted transcriptional and proteomics profiling experiments have produced a comprehensive map of single nucleotide polymorphisms (SNPs), deletions, insertions, and duplications of genomic regions across 14 BCG strains (25, 26). Intriguingly, BCG strains of the duplication group DU2-IV (BCG Phipps, Frappier, Pasteur, and Tice) exhibited the highest levels of virulence in the mouse infection model, whereas BCG strains of the DU2 group II (BCG Sweden and Birkhaug) were among the least virulent group. The more virulent strains were also more effective in protection against *Mycobacterium tuberculosis* challenge (27). At molecular level, BCG-Japan, -Moreau, and -Glaxo strains are naturally defective in the production of phthiocerol dimycocerosates (PDIMs) and phenolic glycolipids (PGLs), two lipid virulence factors, which could compromise their effectiveness during vaccination (28). To compensate for a potential “over-attenuation,” according to the WHO report, a dose of BCG-Japan contains 5-fold more CFU than other distributed BCG vaccines (29). Therefore, selection of the BCG strain, vaccine formulation and route of the administration may ultimately impact the effectiveness of these vaccines against COVID-19 in clinical trials (30).

## BCG and COVID-19

Multiple studies are being carried out around the world in an effort to associate the beneficial effects of BCG vaccination on COVID-19. Initial ecological studies established that in countries with BCG vaccination programs have less COVID-19 cases and deaths per population (31, 32), suggesting that trained immunity inducing vaccines may provide protection to bridge the gap before a COVID-19-specific vaccine is developed (33). On the contrary, other studies concluded that BCG vaccination in childhood does not have a protective effect against COVID-19 in adulthood (34). It is possible that due to the different testing and notification approaches, case and deaths incidences of COVID-19 might not differ in a country with BCG vaccination as it is critical to include different variables of interest such as age structure, income, rurality, and population density. A National Institutes of Health (NIH) study attempted to correct potential confounding variables among countries, such as access to health, education, and stage and size of the COVID-19 epidemic, which observed a strong correlation between BCG vaccination policy and reduction of morbidity and mortality due to COVID-19 in different European countries (10% of the augmentation in the BCG index was associated with a 10.4% mortality reduction from COVID-19) (35). Similar conclusions were reached comparing the same geographical area with similar socioeconomic conditions between Spain and Portugal, with different high mortality rates have been observed in Spain where TB vaccination is no longer part of the official vaccination calendar (36). However, it is important to highlight the importance of including new variants to verify the potential of BCG as a vaccine against COVID-19, such as BCG index (proportion of population of a country vaccinated against BCG), HDI score (number of days since one case per million), population density per Km^2^, population >65 years of age, CPI (Corruption Perception Index, government transparency) and percentage of population living in urban areas.

Only ongoing randomized controlled trials (RCTs) will provide answers to whether BCG reduces the incidence and severity of COVID-19 through its cross-protective effects. The phase III randomized clinical trial ACTIVATE (NCT03296423) confirmed that recent vaccination with BCG in elderly (>65 years) protects against new infections. In this trial in which 198 elderly people participated, it was demonstrated the difference between the incidence of new infections after placebo vaccination (42.3%) and BCG vaccination (25.0%), being most of the protection against respiratory tract infections. Furthermore, vaccinated individuals took longer to get infected (16 weeks) than the ones vaccinated with placebo (11 weeks). Further statistical analysis indicated a 79% decreased on the risk of acquiring at least one new respiratory infection in a 12 months period for BCG vaccinated group. These benefits were suggested to be mediated by pro-inflammatory cytokines, such as IL-10 TNFα and IL1-β, and therefore associated with the induction of trained immunity. Supporting this view, all BCG vaccinated patients showed an increased proinflammatory pattern after a second stimulation of peripheral blood mononuclear cells (PBMCs) with heat-killed *C. albicans* or LPS, although insufficient data was obtained in order to correlate the effect (16). The study concluded that BCG vaccination is safe, as recently reported by the same group (17), and can protect the elderly against infections. The study also suggests that BCG vaccination may be able to protect health workers or vulnerable individuals against SARS-CoV-2 virus infection, although larger and specific studies are needed to assess BCG protection against COVID-19.

Whereas most of the randomized clinical trials are set out to investigate the efficacy in COVID-19 prevention, a BATTLE trial in Brazil (NCT04369794) is designed to test BCG in a therapeutic vaccination setting. Specifically, BATLE trial enrolls patients with confirmed COVID-19 symptoms. This prospective, randomized, double-blind study is testing the potential of BCG to affect (a) clinical evolution of COVID-19, (b) elimination of SARS-CoV-2 at different times and disease phenotypes, and (c) seroconversion rate and titration (anti-SARS-CoV-2 IgA, IgM, and IgG). Such therapeutic vaccination strategy addresses the hypothesis that the induction of both innate and viral-specific immune responses might be beneficial during active SARS-CoV-2 antigenic exposure and provides further rationale for combining BCG-based vaccines with other approved vaccines. Recent preclinical evidence corroborated this notion: BCG:CoVac, a formulation combining BCG with a stabilized form of the spike (S) protein resulted in the stimulation of SARS-CoV-2-specific antibody and T-cell responses in mice at the levels equivalent to or exceeding responses elicited by current clinical-stage vaccines in the murine models (37). Further demonstrating complex interplay between innate and adaptive immunity, a randomized clinical trial of topical BCG in children having common warts caused by the human papillomavirus, showed 65% complete responses, with no response detected in the control group (38). In conclusion, these preliminary studies suggest that BCG vaccination could enhance vaccine induced immunity against SARS-CoV-2.

## Novel BCG-Based Approaches

Unlike other vaccination platforms, the robust safety and immunogenicity profile of BCG has rendered it an attractive vector for vaccine development against many viral or bacterial diseases. Antigens expressed by recombinant BCG (rBCG) strains can elicit long-lasting humoral and cellular immunity, including CD4^+^ and CD8^+^ T cell responses, to the foreign antigens in animals or humans. Recombinant BCG technology has been studied in the context of vaccination against HIV (39, 40), HCV (41), hMPV (42), RSV (43), rotavirus (44), *Bordetella pertussis* (45), Lyme disease (46), malaria (47), and measles (48). Furthermore, when administered in early life, BCG vaccination can act as an adjuvant enhancing antibody responses to recombinant hepatitis B surface antigen (rHBsAg) both in mice and in human infants (49, 50).

One of the prominent novel approaches is the development of live rBCG engineered to express SARS-CoV-2 antigens to enhance innate and adaptive immune responses and induce sustained antigen presentation. The overall design strategy is based on the hypothesized ability of rBCG-SARS-CoV-2 bacteria to deliver antigens to lymphoid organs, prime a polyfunctional T-cell response and induce long-term systemic and pulmonary protective T-cell immunity. Overall, T-cell responses against the S, N, and M antigens have been reported to be the most dominant and long lasting (51). Accordingly, a priority should be given to the viral antigens or epitopes that are identical to the SARS-CoV-1 and do not include any mutations in the available SARS-CoV-2 sequences, specifically mapped to the S, N, or M structural proteins. Several groups around the world are currently pursuing the construction of such next-generation BCG-based vaccines (52, 53). An earlier-generation genetically modified rBCG-based vaccine, VPM1002 has already entered clinical trials (NCT04387409, NCT04439045) for reducing SARS-CoV-2 infection rate and COVID-19 severity. VPM1002 expresses listeriolysin O (LLO) derived from *Listeria monocytogenes* and deleted Urease C (ureC) gene and has been demonstrated to be safer and more immunogenic in preclinical studies (54).

A different approach by Carlos Martin and colleagues generated a live attenuated *M. tuberculosis* vaccine (MTBVAC) that confers protection against pneumonia through the induction epigenetic and metabolic reprograming of monocytes associated with trained immunity (55). This represents a unique approach that does not use a cow-derived attenuated M. bovis strain but a human-derived *M. tuberculosis* with deletion of phoP and faD25 genes, which is currently under clinical trial.

## Discussion

Bacillus Calmette-Guérin (BCG) vaccination has been effective for nearly a century against Tuberculosis (TB), but it has also been described to reduce childhood mortality in a non-specific manner (6, 56, 57). The BCG is a live-attenuated vaccine that represents the most widely used vaccine in the world, with more than 4 billion people vaccinated with BCG worldwide and another 100 million newborn children vaccinated with BCG each year, providing over 50% protection against lung respiratory diseases and over 80% protection against disseminated TB (8). The mechanisms by which BCG induces a cross-protection against other diseases and specifically respiratory tract infections, has been stablished during the last decade and suggests a critical role of the innate immune system (58). The concept of trained immunity brings new insights into the immunological memory concept and described that cells from the innate immune system, such as macrophages, are able to recall a first encounter with a pathogen and produce an enhanced response toward a second assault. As a result, several studies on the cross-protective effects of the BCG vaccines have demonstrated that the positive effects on susceptibility to viral respiratory infections is associated with induction of trained immunity.

Introduction of BCG in developed countries is associated with reduction of mortality that cannot not be explained by a specific disease protection. A recent case cohort study by Aaby et al. reported a better survival rates in Denmark associated to smallpox and tuberculosis vaccination during childhood (59). The study described that individuals who were vaccinated at a young age with both BCG and Vaccinia had a lower mortality rate (adjusted hazard ratio of 0.54) compared to non-vaccinated individuals (adjusted hazard ratio of 1). Interestingly, the study demonstrated the protective role of BCG to lower natural causes of death (adjusted hazard ratio of 0.57) after more than 30 years since BCG vaccination, suggesting that BCG may contribute to lower the mortality long-term. Together with other studies, these conclusions led to further investigations aimed at determining the durability of the BCG vaccine. A similar study suggested that the BCG vaccine remains effective after several decades following vaccination. A retrospective population-based cohort study carried out in Norway revealed that the BCG vaccine effectiveness against pulmonary tuberculosis remains at 40% after 30–40 years (60). Since BCG-derived immunity persist beyond 15 years after vaccination, it is plausible to hypothesize that BCG-induced non-specific protection could also last for decades. Consistent with this view, Mayda and Ishan Gursel hypothesized that countries with continuing BCG immunization programs better contain the spread of SARS-CoV-2 and reported statistical differences between five European countries after 11–22 years since last BCG vaccination and 8 European countries after 30–45 years. These studies also speculate that BCG re-vaccination represents a valid approach to increase the vaccine effectiveness (31). On the other hand, other studies demonstrated the absence of correlation between a decrease of positive SARS-CoV-2 test results and BCG-vaccinated adults aged 39–41 years vs. non-vaccinated aged 35–37 years, suggesting that the BCG vaccine does not interfere with infection in young adults (61). A recent retrospective observational study carried out in healthcare workers in Los Angeles demonstrated that history of BCG vaccination was associated with an altered seroprevalence and infection with SARS-CoV-2. Specifically, the study indicated that BGC vaccinated healthcare workers were less likely to suffer COVID-19 related symptoms (fatigue, dry cough, and muscle aches), were associated to have a reduced rate of testing positive either with a COVID-19 diagnosis by a medical doctor or a SARS-CoV-2 RT-PCR test and had a significantly lower positive serology against SARS-CoV-2 (IgG) (62). Overall, these finding suggest that BCG vaccination may mitigate sickness associated with COVID-19 infection and ongoing clinical trials are aimed at specifically address this question.

Since the BCG vaccine has proven protection against viral respiratory infections, several laboratories are exploring the possibility that the BCG vaccine could be used alone or synergistically to reduce COVID-19 disease severity. Nowadays, around 20 controlled randomized clinical trials ongoing in the Netherlands, Australia, Germany, Greece, the United States, Egypt, Colombia, Mexico, Brazil, South Africa, Denmark, and France (Table 1) to evaluate whether the BCG vaccine decreases the incidence and the severity of COVID-19. The importance of establishing age groups that might be protected against SARS-CoV2 infection by BCG-vaccination is evident, as well as the timing of the vaccine before potential exposure to the virus, as this may determine whether the BCG-vaccine is effective. This represents a critical step to assess the potential of BCG-vaccination to protect the elderly against SARS-CoV-2 and other respiratory infectious agents. However, additional from the above ongoing RCTs are needed to specifically demonstrate the effects of BCG vaccination in the morbidity and mortality of COVID-19 in different scenarios. For example, BCG could be administered in clinics via multiple routes, which potentially include direct mucosal delivery. Whereas current trials with BCG for COVID-19 all rely on the intradermal injections, the oral BCG has been used in Brazil for vaccination against TB until the 1970s. Mimicking the natural mycobacterial infection route by mucosal vaccination with BCG has been known to generate superior protection against TB in animal models. Oral or intranasal BCG vaccination has been shown to induce greater mucosal immune responses and better protection against pulmonary TB compared with subcutaneous vaccination in animals via potent induction of lung parenchyma- and airway-resident memory T cells populations (63, 64). In this respect, immunization with recombinant BCG-N-hRSV protects from hRSV virus associated-lung damage, decreases the infiltration of inflammatory immune cells to the lungs and reduces the virus in the lung tissues when mice were infected with hRSV (65, 66). Based on this evidence, it is possible that mucosal (oral or aerosolized) administration of BCG that engages trained immunity locally may lead to more effective innate immune memory responses in lungs of COVID-19 patients.

**Table 1:** Clinical trials selected from clinicaltrials.gov and additional European studies from the WHO ICTRP.

**Trial ID**	**Source**	**Acronym**	**Status**	**Interventions**	**Type**	**Design**	**Phase**	**Primary**	**Enrollment**	**Age**	**Locations**	**Strain**
NCT04327206	ClinicalTrials.gov	BRACE	Recruiting	BCG vaccine vs. 0.9%NaCl	Interventional	Randomized	3	Prevention	10,078	≥18 years	Australia	Danish strain 1331
NCT04369794	ClinicalTrials.gov	BATTLE	Recruiting	BCG vaccine vs. placebo	Interventional	Randomized	4	Treatment	1,000	≥18 years	Brazil	N/A
NCT04659941	ClinicalTrials.gov	ProBCG	Recruiting	BCG vaccine	Interventional	Randomized	2	Prevention	1,000	≥18 years	Brazil	N/A
RBR-4kjqtg/U1111-1256–3892	REBEC		Recruiting	BCG vs. placebo	Interventional	Randomized	2	Prevention	800	≥18 years	Brazil	Moscow strain 361–1
NCT04439045	ClinicalTrials.gov	COBRA	Recruiting	VPM1002 vs. placebo	Interventional	Randomized	3	Prevention	3,626	≥18 years	Canada	VPM1002
NCT04641858	ClinicalTrials.gov	EDCTP	Recruiting	BCG-Denmark vs. saline	Interventional	Randomized	4	Prevention	1,050	≥18 years	Cape Verde, Guinea-Bissau, Mozambique	Danish strain 1331
NCT04373291	ClinicalTrials.gov		Recruiting	BCG-Denmark vs. saline	Interventional	Randomized	3	Prevention	1,500	18–100 years	Denmark	Danish strain 1331
NCT04542330	ClinicalTrials.gov		Recruiting	BCG-Denmark vs. saline	Interventional	Randomized	3	Prevention	1,900	65–110 years	Denmark	BCG-1331, AJ Vaccines
NCT04347876	ClinicalTrials.gov		Recruiting	Diagnostic Test: Tuberculin test	Observational	Case-Control			100	12–80 years	Egypt	N/A
NCT04350931	ClinicalTrials.gov		Not yet recruiting	BCG vaccine vs. placebo	Interventional	Randomized	3	Prevention	900	≥18 years	Egypt	Danish strain 1331
NCT04384549	ClinicalTrials.gov	COVID-BCG	Recruiting	BCG vaccine vs. placebo	Interventional	Randomized	3	Prevention	1,120	≥18 years	France	N/A, AJ Vaccine
NCT04387409	ClinicalTrials.gov		Active, not recruiting	VPM1002 vs. placebo	Interventional	Randomized	3	Prevention	59	≥18 years	Germany	VPM1002
NCT04435379	ClinicalTrials.gov		Recruiting	VPM1002 vs. placebo	Interventional	Randomized	3	Prevention	2,038	≥60 years	Germany	VPM1002
NCT04414267	ClinicalTrials.gov	ACTIVATEII	Recruiting	BCG vaccine vs. placebo	Interventional	Randomized	4	Prevention	900	≥50 years	Greece	Moscow strain 361-1
EUCTR2020-001783-28-HU	EU Clinical Trials Register	BACH	Authorized	BCG vs. placebo	Interventional	Randomized	3	Prevention	1,000	≥18 years	Hungary	Danish strain 1331, SSI, Denmark
NCT04475302	ClinicalTrials.gov		Recruiting	BCG vaccine (Freeze-dried)	Interventional	Non-Randomized	3	Prevention	2,175	60–80 years	India	N/A, Serum Institute of India
CTRI/2020/06/ 025854	CTRI		Not Recruiting	BCG vaccine	Interventional	Non-randomized	N/A	Prevention	1,450	60–95 years	India	BCG-Serum Institute of India
CTRI/2020/04/ 024833	CTRI		Not Recruiting	BCG vs. placebo	Interventional	Randomized	N/A	Prevention	1,826	18–65 years	India	BCG-Denmark, Green Signal
CTRI/2020/05/ 025013	CTRI		Not Recruiting	BCG plus STANDARD of CARE as suggested by DCGI vs. SALINE plus STANDARD of CARE as suggested by DCGI	Interventional	Non-randomized	2	Treatment	60	20–50 years	India	Tubervac (Serum Institute of India)
CTRI/2020/09/ 027684	CTRI		Recruiting	BCG vaccine vs. BCG re-vaccination	Interventional	Randomized	N/A	Prevention	400	18–50 years	India	N/A
CTRI/2020/04/ 024749	CTRI		Not Recruiting	VPM1002 vs. placebo 0.9% saline	Interventional	Randomized	3	Prevention	5,946	18–99 years	India	VPM1002
CTRI/2020/07/ 026668	CTRI		Not Recruiting	BCG vs. placebo	Interventional	Randomized	3	Prevention	800	18–60 years	India	N/A
IRCT20200411 047019N1	IRCT		Recruiting	BCG vs. placebo	Interventional	Randomized	3	Prevention	500	≥18 years	Iran	N/A
NCT04461379	ClinicalTrials.gov		Active, not recruiting	BCG vaccine vs. placebo	Interventional	Randomized	3	Prevention	908	≥18 years	Mexico	Tokio 172
NCT04328441	ClinicalTrials.gov	BCG-CORONA	Active, not recruiting	BCG vaccine vs. placebo	Interventional	Randomized	3	Prevention	1,500	≥18 years	Netherlands	Danish strain 1331
NCT04417335	ClinicalTrials.gov		Active, not recruiting	BCG vaccine vs. placebo	Interventional	Randomized	4	Prevention	2,014	≥60 years	Netherlands	Danish strain 1331
NCT04537663	ClinicalTrials.gov	BCG-PRIME	Recruiting	BCG vaccine vs. placebo	Interventional	Randomized	4	Prevention	5,200	≥60 years	Netherlands	Danish strain 1331
EUCTR2020-002456-21-NL	EU Clinical Trials Register	BCG-PLUS	Not Recruiting	BCG, BCG plus MMR, BCG plus alendronic acid, alendronic acid, placebo	Interventional	Randomized	4	Prevention	100	18–64 years	Netherlands	N/A, AJ VACCINES
NL8547	Netherlands Trial Register	BCG-CORONA-ELDERLY	Recruiting	BCG vs. placebo	Interventional	Randomized	N/A	Prevention	1,600	≥60 years	Netherlands	N/A
NCT04648800	ClinicalTrials.gov		Recruiting	BCG-10 vaccine vs. 0.9% saline	Interventional	Randomized	3	Prevention	1,000	≥25 years	Poland	Moreau
NCT04379336	ClinicalTrials.gov		Recruiting	BCG vaccine vs. placebo	Interventional	Randomized	3	Prevention	500	≥18 years	South Africa	Danish strain 1331
NCT04348370	ClinicalTrials.gov	BADAS	Recruiting	BCG vaccine vs. placebo	Interventional	Randomized	4	Prevention	1,800	18–75 years	United States	Tice BCG
NCT04534803	ClinicalTrials.gov		Not yet recruiting	BCG vaccine vs. placebo	Interventional	Randomized	3	Prevention	2,100	≥70 years	United States	Tokio 172
NCT04632537	ClinicalTrials.gov	NUEVA	Recruiting	Tice BCG vs. saline	Interventional	Randomized	3	Prevention	550	18–64 years	United States	Tice BCG

We conclude that, in addition to protection from COVID-19 disease, SARS-CoV-2 viral production and death, BCG clinical trials that evaluate (i) alternative routes of administration, (ii) its potential use as adjuvant, and (iii) its potential to prevent concurrent respiratory diseases will be very informative. While some vaccines rely on additional adjuvants in their formulation, live BCG has strong “self-adjuvant” properties that stimulate multiple innate immune sensors or PRRs, including TLR2, TLR4, TLR8, C-type lectin receptors Dectin-1, and Mincle that enhance vaccine induced immunity (67). While the main line of research and therapeutic approaches are focused on the adaptive immune response against SARS-CoV-2, we should not forget the primordial role of the innate immune response against infections. The possibility to stimulate the innate immune system represents a synergistic methodology that should be further explored to shield individuals against SARS-CoV-2 and unknown invading pathogens. Together with BCG, other live attenuated vaccines are currently used to protect against specific infections such as vaccinia (MVA), YFV, measles, mumps, rubella, rotavirus, or attenuated influenza vaccines. It will be therefore interesting to explore the effects of these vaccines that trigger the innate immune response to enhance the basal defenses against coming pathogens, with open questions about their specificity, level of protection, and durability (68).

## Author Contributions

All authors listed have made a substantial, direct and intellectual contribution to the work, and approved it for publication.

## Conflict of Interest

The authors declare that the research was conducted in the absence of any commercial or financial relationships that could be construed as a potential conflict of interest.
